# Effects of short duration static stretching on jump performance, maximum voluntary contraction, and various mechanical and morphological parameters of the muscle–tendon unit of the lower extremities

**DOI:** 10.1007/s00421-014-3047-y

**Published:** 2014-11-16

**Authors:** Savvas Stafilidis, Markus Tilp

**Affiliations:** Institute of Sports Science, Graz University, Mozartgasse 14, 8010 Graz, Austria

**Keywords:** Static stretching, MVC, Stiffness, Strain, Ultrasound, Jump performance

## Abstract

**Purpose:**

Static stretching is used in sport practice but it has been associated with decrements in force and performance. Therefore, we examined the effect of short duration static stretch on the mechano-morphological properties of the m. vastus lateralis (VL) muscle tendon unit (MTU) and on the jumping performance.

**Methods:**

Eight males and three females (mean ± SD, 25.5 ± 3.1 years) stretched their lower legs for a 15 or 60 s duration or acted as their own control without stretching in a randomized order. In a pre-post design, a passive movement (5°/s) and a maximum voluntary knee extension contraction (MVC) were performed on dynamometer while the VL tendon and aponeurosis was observed via ultrasound. Furthermore, the participants performed countermovement (CMJ) and squat jumps (SJ).

**Results:**

Repeated measures ANOVA did not show significant differences in MVC, active and passive strain, stiffness, elongation, knee joint angle range, and jump performance between and within groups.

**Conclusions:**

The applied stretch stimuli (15 or 60 s) were not sufficient to trigger adaptations in the mechano-morphological properties of the lower extremities MTU which therefore did neither affect jump performance nor MVC. As a possible mechanism, we hypothesized that the dose-time dependency effect of static stretch might have important implications when measuring functional parameters of the MTU and performance. Further examination is necessary to elucidate its impact in the examination of the MTU mechano-morphological properties.

## Introduction

Static stretching is an essential part of recreation or competitive sports activities as well as for rehabilitation treatment (Beckett et al. 2009; Nelson et al. 2005; Wu et al. 2011; Pin et al. 2006; Zhao et al. 2011). Among other factors like intensity and frequency (Herda et al. 2011; Ryan et al. 2009) researchers showed that different durations of static stretches have diverse effects on muscles, tendons, and the neural response on the muscles (Ryan et al. 2008a; Winchester et al. 2009). Furthermore, various studies reported that static stretching can reduce passive resistive torque (Kay and Blazevich 2009a), lower the maximal exerted peak torque (Fowles et al. 2000), or impair muscular performance (Behm and Kibele 2007; Power et al. 2004). Researchers proposed several possible mechanisms responsible for the diminishing effects of static stretching on force development. While Avela et al. (1999) and Guissard et al. (2001) showed indices of decreased motor neuron excitability, Cramer et al. (2005) confirmed the neural origin of the stretch-induced strength loss on the non-stretched contralateral limb. Alterations in the mechanical properties of the muscle tendon unit were also reported as possible mechanism for the strength reduction after passive stretch (Fowles et al. 2000; Weir et al. 2005). Furthermore, researchers examined the effect of the stretch-induced strength loss on various stretch shortening activities and found detrimental impact on performance. Beckett et al. (2009) indicated that the repeated sprint ability was negatively affected by an interim static stretching intervention with a total duration of ~4 min. Furthermore, Nelson et al. (2005) examined the effect of a 4 × 30 s pre-exercise static stretching on sprint performance and found a significant increase of the examined 20 m sprint time (decrease in performance). In another study (Young and Elliott 2001) showed that a 3 × 15 s static stretching intervention of the lower extremities was able to induce a decrease in the drop jump performance but not on the concentric explosive muscle performance. In another study, Behm and Kibele (2007) applied a 4 × 30 s static stretch on the lower extremities with different intensities ranging from 50 to 100 % of the point of discomfort (POD). In all examined conditions with repetitive short stretches accumulating to moderate stretching times, (short <90 s total, moderate >90 s total, definition according to Behm and Chaouachi 2011) the authors found a decrease in performance ranging from 3.6 to 5.7 % in various stretch–shortening jump tests and suggested that possible alterations in the muscle compliance could play a role in those performance diminishing effects. Opposing to the aforementioned studies, others reported that a moderate bout of static stretch (4 × 45 s) does not have negative impact on the force production capability or the muscle tendon mechanical properties (Cannavan et al. 2012). Furthermore, after applying a 270 s static stretch to the muscle groups of the examined dominant leg Power et al. (2004) found a decrease of torque development or force produced voluntarily and the muscle inactivation as measured by the interpolated twitch technique (ITT). However, this was not found in the subsequently examined one leg drop and squat jump.

In contrast to studies with moderate stretching time, most of the studies with short overall static stretch duration (5–60 s) found different results. For example in a dose–response study, Kay and Blazevich (2008) examined the effect of small static stretch durations (5, 15, 4 × 5 and 4 × 15 s) on passive and peak isometric plantar flexor moment. The authors found a decrease in the force production which was significantly correlated (*r* = 0.68, *p* < 0.01) to the applied stretch duration. However, a significant decrease in force compared to the control group was only apparent after the 4 × 15 s stretch duration. Therefore, the authors pointed out that the amount of force loss is duration-dependent but neither the stiffness properties of the muscle tendon complex or the neural excitability could explain those differences. Pinto et al. (2014) examined the effect of 30 and 60 s of static stretch on the jump performance by means of CMJ. Compared to the control group the authors found a significant negative effect of the 60 s duration static stretch on jump performance (−3.4 %, *p* < 0.05), as well as on average (−2.7 %, *p* < 0.05) and peak power output (−2 %, *p* < 0.05). However, the 30 s duration showed no significant difference to other conditions. Therefore, the authors concluded that there is a threshold in the static stretch duration where a multi-joint task (CMJ), when practiced immediately after, can be negatively affected. On the contrary, Fortier et al. (2013) examined the acute effects of short duration (20 s) isolated static stretch of the lower extremities with and without dynamic plyometric exercises on strength, jumping, and sprinting. The authors found a significant decrease only in the jump performance (−4 %, *p* < 0.05), but not in the other parameters indicating that short duration static stretch prior to a dynamic task is not an efficient method to increase performance. However, in this study even the non-stretching control group showed a significant decrease in CMJ height, indicating that the stretch intervention might not be the causal reason for the decrease. Winchester et al. (2009) demonstrated that a single static stretch maneuver (30 s) is sufficient to significantly reduce the maximal exerted force of the hamstrings muscles.

Several authors (Young and Behm 2003; Behm and Chaouachi 2011; Simic et al. 2013; Young and Elliott 2001) recommended reduced duration or complete avoidance of static stretching prior high explosive movements based on findings that indicated diminishing outcomes on speed (Beckett et al. 2009); jumping performance (Behm and Kibele 2007; Young and Elliott 2001) or rate of force development (McBride et al. 2007). However, according to the literature the applied mean static stretching time in one muscle group in various competitive sport activities does not exceed 18 s (Ebben and Blackard 2001; Simenz et al. 2005; Ebben et al. 2005). Thus, the majority of the studies which examined the effect of static stretch on muscle performance used stretching protocols that exceeded the actual duration of static stretch implemented in sport practice.

Therefore, it is reasonable to challenge the recommendations supporting the reduction or even the absence of static stretching in the pre-exercise phase in order to avoid possible negative effect on performance. Hence, the main goal of this study is to investigate the influence of short duration static stretch of the lower extremities on the force production ability and its effect on the jump performance and mechano-morphological properties of the vastus lateralis (VL) tendon and aponeurosis. Based on previous literature findings we hypothesized that a small duration (15 s) of static stretch as practiced in competitive sports activities would not alter the mechano-morphological properties of the VL tendon and aponeurosis and would not have negative impact either on muscle force or the jumping performance. Contrariwise, we expected contrasting results when the stretch duration exceeds 45 s of stretch (Simic et al. 2013).

## Methods

### Experimental design

Eleven students of the university population participated in this study. Eight males and three females (mean, SD: age 25.5 ± 3.1 years; height, 176.2 ± 7.5 cm; body mass 73 ± 7.5 kg) completed all experimental conditions and acted as their own control with a 24 h time gap between sessions. All participants were recreational athletes from various sports activities performing approximately 7 h training per week and familiarized with stretching and jump exercises. Written informed consent was obtained prior to participation, and subjects were informed that they could withdraw at any time. The study was approved by the Ethical Committee of the University of Graz and conformed to the guidelines of the declaration of Helsinki. All participants were familiarized with the test procedure performing several knee extension contractions and jumping tasks prior to the experiment. All participants were requested to abstain from strenuous exercise 48 h before data collection.

A 5 min warm-up on a cycle ergometer at 60 RPM was used to increase the body temperature prior to the test. Subsequently, the participants were placed on the dynamometer (Con-Trex Multi Joint, CMV AG, Duebendorf, Switzerland) where the knee joint was carefully aligned to the dynamometer axis shaft. The body (hip joint 110°) was tightly secured with inextensible belts to prevent it from slipping. An adhesive tape stripe (5 mm width) was placed approximately at the two-third distance of the epicondylus lateralis to the trochanter major on the Vastus Lateralis (VL) in a medio-lateral orientation. The knee joint was set to an angle of 115° (180° is fully extended) at which position the force acting on the VL tendon and aponeurosis is zero (Riener and Edrich 1999). To assess the resting length of the VL tendon and aponeurosis we measured the distance from the tuberositas tibiae to the stripe on the VL with an inelastic measuring tape.

The knee joint range of motion was individually set from the point of maximal knee flexion to the maximum knee extension. The dynamometer range of motion setting was identical in pre and post measurement and was readjusted for every participant and condition. Gravity corrections were performed before each isometric measurement trial. All preparations and settings were conducted by the same researcher in order to avoid random errors.

### Range of motion and passive resistive torque

The investigation of the passive range of motion was conducted by using the isokinetic dynamometer. The participants were seated on the dynamometer and the knee joint rotation was manually controlled from the individual full extension to the maximum achievable knee joint flexion. Three consecutive cycles at 5°/s were performed and measurements were attained at the last round in order to avoid preconditioning effect (Taylor et al. 1990). Furthermore, the participants were asked to avoid any muscle tension during the measurements. Visual control of the muscle fascicles on the ultrasound monitor was used (Karamanidis et al. 2011) to identify and exclude trials from further analysis when the subject's VL muscle evidently was not in a relaxed state.

**Fig. 1:**
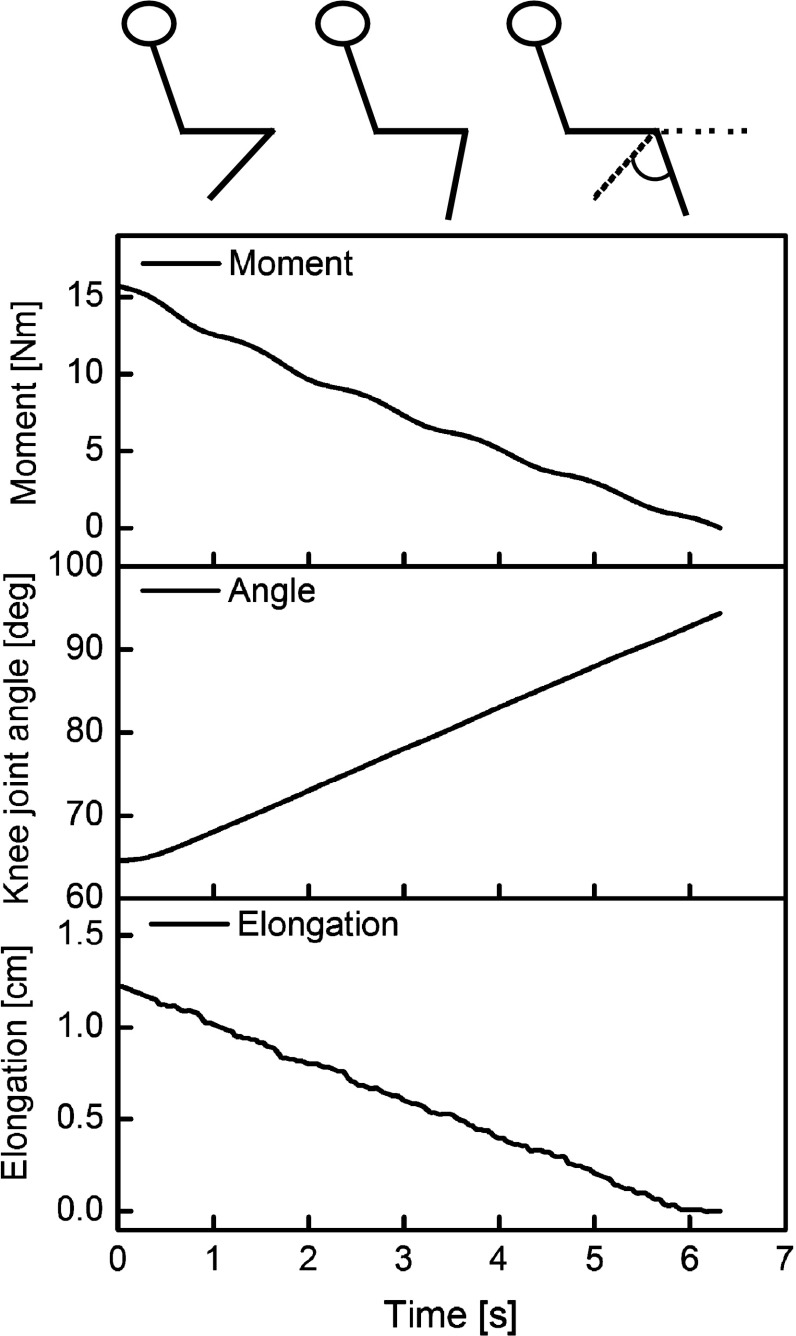
Example from one participant’s passive exerted moment, knee joint angle alteration and VL tendon and aponeurosis elongation over time. Stick figure is showing the knee joint angle change and the knee joint angle range. Straight leg was defined as 180°

Passive resistive torque was measured between the maximum achievable knee flexion (mechanical restraint) position and the knee angle where the knee joint exerted zero torque (~115° knee joint angle). The difference between maximal knee flexion and the knee joint angle where exerted torque was zero was defined as knee joint angle range (Fig. 1). The experimental setup did not allow for the knee joint to fully flex due to the mechanical constraints produced by the position of the participant on the dynamometer. Since the maximal achievable knee flexion angle remained constant in the pre and post trial we could also measure any possible passive mechanical alteration of the VL tendon and aponeurosis as a shift in the angle where the knee joint exerted zero torque (torque/angle shift).

### Maximal voluntary contraction

**Fig. 2:**
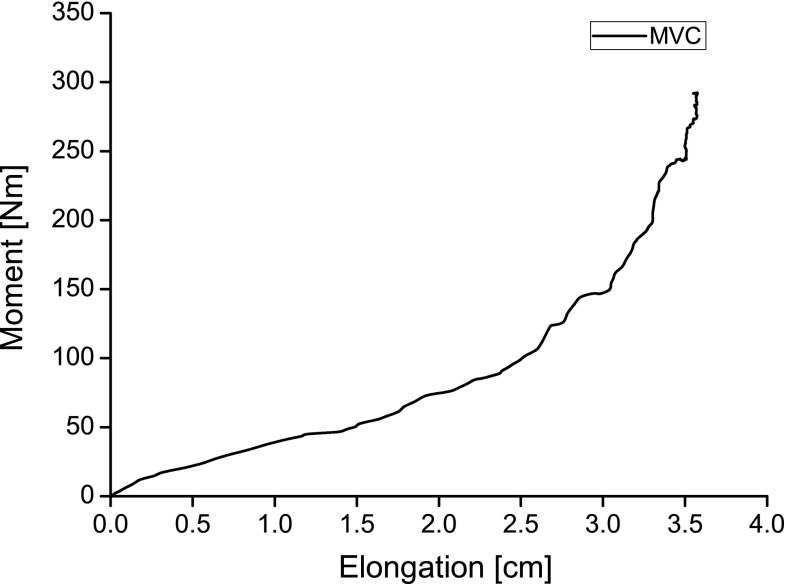
Example from one participant’s MVC exerted knee extension moment and VL tendon and aponeurosis elongation

Preceding the MVCs the participants performed 2 and 1 submaximal ramp isometric voluntary contractions in the pre and post session respectively to prevent any preconditioning effect (Maganaris 2003) with a rest period of 3 min between efforts. The participants were provided with visual feedback during the submaximal voluntary contractions by displaying the achievable torque on a monitor. The knee joint angle was set at 120° since in this position the potential of force generating capacity due to the force–length relationship is maximized (Herzog et al. 1990). The participants executed one voluntary isometric ramp contraction consisting of 5 s increase, 3 s plateau, and 5 s decrease of force (Fig. 2). Verbal encouragement was given during every effort. All data were recorded in a portable computer device at 1,000 Hz and stored for further analysis. The kinetic data were filtered with a fourth order zero-lag low pass Butterworth filter with a cutoff frequency of 1.5 and 10 Hz for the passive and MVC trials, respectively.

### Ultrasonography measurements

To obtain the longitudinal image of the VL, we used a real-time ultrasound apparatus (mylab 60, Esaote S.p.A., Genova, Italy) with a 10 cm B-mode linear-array probe (LA 923, Esaote S.p.A., Genova, Italy, 10 MHz) at 35 Hz. The ultrasound transducer was placed on the mid part of the VL over the adhesive stripe. To prevent the ultrasound probe from slipping during the MVC and passive trials, we placed it in a custom-built case of Styrofoam and secured it with elastic bands. We synchronized the kinetic data with the ultrasound images with a custom-built manual trigger apparatus which gave simultaneously a 5 V signal on the measuring computer, and it was displayed as a spike line on the ultrasound images.

**Fig. 3:**
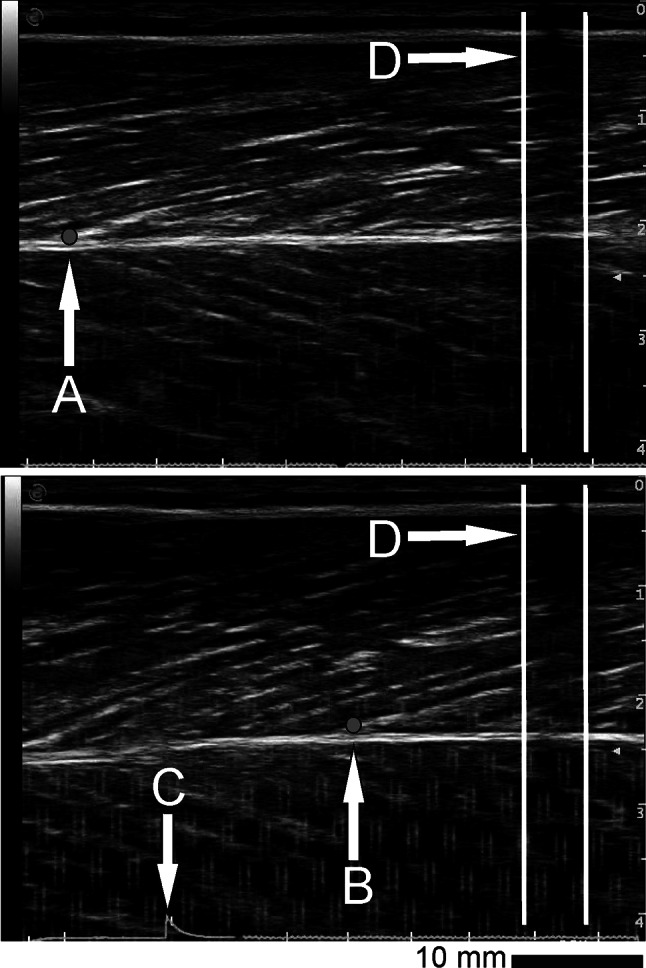
VL fascicle insertion point on the deep aponeurosis at rest *upper image* (**a**), and during an isometric MVC *lower image* (**b**). Synchronization signal is displayed as spike in the bottom of the image (**c**). We calculate the displacement of the insertion point in relation to the skin marker here visible as vertical hypoechoic area (**d**)

Ultrasound images sequences were internally recorded at 25 Hz, cut and digitized by VirtualDub open-source software (version 1.6.19, http://www.virtualdub.org). The ultrasound echo of the fascicle insertion to the deep aponeurosis was manually tracked and stored with open source video analysis software (Tracker 4.84, https://www.cabrillo.edu/~dbrown/tracker/). In order to obtain the elongation of the VL tendon and aponeurosis the displacement of the fascicle insertion point on the deep aponeurosis was measured relative to the skin marker placed over the VL belly (Fig. 3). Therefore, the elongation of vastus lateralis tendon and aponeurosis represents the elongation of all structures distal to the analyzed point. The reproducibility of this method was established in previous studies (Bojsen-Møller et al. 2003; Maganaris and Paul 2000). To achieve a common frequency, we interpolated the VL tendon and aponeurosis elongation data at 1,000 Hz using a cubic spline function. The calculated stiffness of the VL tendon and aponeurosis was defined as the slope of the linear regression fitted from 50 to 100 % of the active torque–elongation relationship (Kubo et al. 2001). Similarly, an estimate for passive stiffness was calculated from 0 to 100 % for the passive trials. Strain was defined as the length change of the tendon and aponeurosis of all structures distal to the analyzed points to the rest length × 100 (Stafilidis and Arampatzis 2007). All calculations were processed by using the MATLAB 2013b (Math Works, Inc., Natick, MA) software packet.

### Vertical jumps

**Fig. 4:**
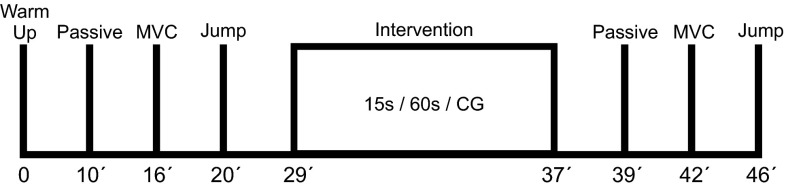
Diagram with the time intervals of the work flow for the measuring and intervention task. After completion of the warm-up passive, active and jump trials were conducted to assess the mechano-morphological properties of the MTU and overall performance. Overall static stretch intervention or control lasted for ~8 min and subsequently the same measurement protocol was repeated

The maximal overall dynamic performance was tested by means of squat and counter movement jumping tasks on a mobile force platform (Quattro Jump, Kistler, Switzerland, 500 Hz). Both jumping tests were conducted prior and following the applied stretch regime (Fig. 4) and the passive and MVC mechano-morphological assessment. The participants were instructed to perform three jumps with maximum effort and as high as possible. In all jumping tasks the participants were required to hold their hands on the hips during the movement. Visual control was carried out to identify failing attempts, and in case of a fault trial the participants were required to repeat the task. For the squat jump, the participants were directed to bend their knees to ~90° and attend that position for at least 3 s prior to the jump. In case of counter movements, trials were automatically excluded from analysis and the jump was repeated.

For the countermovement jump the participants were guided to stand still in a straight position for 3 s prior to the jump on the force platform. A trial was assumed invalid when the hands were not held to the hip during the whole movement and was excluded from further analysis. Consequently, the trial was repeated.

A rest time of 1 min between trials and 3 min between sets was conducted in order to avoid fatigue. For the statistical analysis we used the mean values of all jump performances that did not vary over 5 %.

### Stretches

The participants stretched the muscle group’s quadriceps femoris, triceps surae, and hamstrings of both legs according to a randomized stretch protocol. For the quadriceps stretch the participants stood upright on one leg and pulled the ankle of the contralateral leg up to the maximum knee flexion. To stretch the hamstrings, the participants laid the leg fully extended on a bench and leaned their upper body forward (hip flexion). For the triceps surae muscle group, the participants leaned against a wall with the front leg bend at ~90° and the rear leg fully stretched. The heel of the stretched leg was kept on the ground for the whole stretching time. Two different time intervals were implemented in order to simulate the stretch duration (15 s) used in the athletic practice and a longer one (60 s) where short-term adaptations were expected (Simic et al. 2013).

All stretches were carried out until the POD and the time intervals of the intervention were kept by the same researcher. Furthermore, the stretches were executed without rest between tasks. The control group remained seated and did not perform any static stretch for a period of 8 min in accordance to the time of the longest stretching treatment (Fig. 4). For the remainder time of the 15 s group, the participants also remained seated.

### Statistical analyses

SPSS (version 21.0, SPSS Inc., Chicago, Illinois) was used for all statistical analyses. A 2 × 3 ANOVA for repeated measurements were conducted in order to determine differences between (a) pre–post and (b) the three (15, 60, CG) stretching conditions. If significant interaction effects (time vs. group) were present, a LSD post hoc analysis was conducted. The level of statistical significance was set to *P* < 0.05 for all comparisons.

## Results

Trials that did not meet scientific standards due to technical complications (primarily due to poor image quality) were excluded from further analysis.

There were no significant (*p* > 0.05) interaction effects between time (pre/post stretch) and stretch duration in the maximal exerted torque (*F*(2,13) = 0.25, *p* = 0.78, effect size = 0.04), elongation (*F*(2,13) = 2.26, *p* = 0.144, effect size = 0.258), stiffness (*F*(2,13) = 0.2, *p* = 0.82, effect size = 0.03), and strain (*F*(2,13) = 1.32, *p* = 0.3, effect size = 0.17). All the results of the MVC trials are displayed in Table 1.

**Table 1:** Elongation, stiffness, strain of the VL tendon and aponeurosis as well as the maximal exerted torque of the m. quadriceps femoris during maximal voluntary contraction

Group: MVC	15 s (*n* = 9)		60 s (*n* = 10)		CG (*n* = 11)	
	Pre	Post	Pre	Post	Pre	Post
Elongation (cm)	2.22 ± 0.50	2.69 ± 0.89	2.38 ± 0.40	2.42 ± 0.81	2.35 ± 0.56	2.37 ± 0.49
Torque (N m)	242.2 ± 46.8	249.0 ± 50.2	236.0 ± 62.5	236.1 ± 68.8	234.8 ± 47.4	232.3 ± 51.3
Stiffness (N m/cm)	164.3 ± 62.6	151.0 ± 56.0	162.3 ± 82.0	152.1 ± 56.2	161.2 ± 77.8	156.6 ± 50.4
Strain (%)	5.9 ± 1.3	7.2 ± 2.1	6.5 ± 1.4	6.7 ± 2.4	6.9 ± 1.7	6.8 ± 1.7

Presented are the three intervention groups in the pre–post test. Values are presented as mean ± SD

Similar to the MVC measurement, we found no significant interaction effects between time (pre/post stretch) and stretch duration (*p* > 0.05) on the passive torque (*F*(2,13) = 0.48, *p* = 0.63, effect size = 0.07), passive elongation (*F*(2,13) = 0.05, *p* = 0.956, effect size = 0.007), estimated stiffness (*F*(2,13) = 0.009, *p* = 0.991, effect size = 0.001), and strain (*F*(2,13) = 0.227, *p* = 0.8, effect size = 0.03). Also the angle range (maximal achievable knee joint flexion to zero torque knee joint angle) showed no significant interaction effect (*F*(2,13) = 0.133, *p* = 0.89, effect size = 0.02) (Table 2).

**Table 2:** Elongation, passive torque, stiffness, strain of the VL tendon and aponeurosis, and angle range of the knee joint during passive resistive trials

Group: PASS	15 s (*n* = 8)		60 s (*n* = 10)		CG (*n* = 11)	
	Pre	Post	Pre	Post	Pre	Post
Elongation (cm)	0.84 ± 0.21	0.88 ± 0.27	0.94 ± 0.29	0.98 ± 0.24	0.83 ± 0.32	0.8 ± 0.2
Moment (N m)	10.8 ± 2.7	10.8 ± 2.8	11.4 ± 2.8	11.0 ± 2.5	9.8 ± 2.4	10.4 ± 2.3
Stiffness (Nm/cm)	12.6 ± 2.7	12.1 ± 2.4	12.2 ± 3	11.3 ± 2.2	12.5 ± 2.5	12.5 ± 2.5
Strain (%)	2.21 ± 0.64	2.28 ± 0.73	2.50 ± 0.81	2.45 ± 0.58	2.2 ± 1.43	2.22 ± 0.93
Knee angle range (°)	25.2 ± 3.8	25.0 ± 4.6	27.2 ± 4.5	26.6 ± 3.7	23.3 ± 3.8	23.4 ± 3.6

Presented are the three intervention groups and the pre–post test. Values are presented as mean ± SD

Also, at both jumping tasks (Table 3) the statistical analysis showed no significant (*p* > 0.05) interaction effect for the SJ (*F*(2,19) = 0.48, *p* = 0.63, effect size = 0.048) and for the CMJ (*F*(2,19) = 0.66, *p* = 0.53, effect size = 0.065).

**Table 3:** Results of the squat and counter movement jump of the three intervention groups and the pre–post test

Group: task	15 s (*n* = 11)		60 s (*n* = 11)		CG (*n* = 11)	
	Pre	Post	Pre	Post	Pre	Post
SJ (cm)	41.8 ± 6.3	41.7 ± 6.3	42.3 ± 6.4	41.7 ± 6.5	42.5 ± 6.4	41.5 ± 6.8
CMJ (cm)	43.0 ± 6.6	43.4 ± 8.1	43.9 ± 6.2	43.6 ± 6.9	44.2 ± 7.3	42.5 ± 6.9

Values are presented as mean ± SD

The calculated linearity (*r* ^2^) of the MVC stiffness lay between 0.94 and 0.98 at the 50–100 % region of the torque–elongation relationship and between 0.96 and 0.97 for the passive trials at the 0–100 % range for all groups in the pre and post trials.

## Discussion

### Main finding

The major finding of the present study is that an acute static stretch regime of 15 or 60 s did neither alter the isometric muscle force production nor had affected the VL tendon and aponeurosis mechano-morphological properties. Furthermore, the jumping performance did not show any statistical difference between and within groups indicating that the stretch duration utilized in the present study was not sufficient to trigger a short-term adaptation process on the force generating mechanism or the series elastics elements which could influence the jumping performance.

### Maximal voluntary contraction

The absence of differences in the MVC presented here is in accordance to the study of Cannavan et al. (2012) where a comparable static stretch regime (3 min) of the plantar flexors did not lead to an effect on the maximal exerted force, the rate of force development, and the tendon elasticity. The authors pointed out that moderate static stretching does not always impair the muscle ability to generate maximal or rapid force. Similar Ryan et al. (2008b) stretched passively for 2–4 and 8 min in their study and examined the dose and temporal response of stretching on peak isometric torque, the percent voluntary activation (%VA), EMG amplitude, peak twitch torque (PTT), rate of twitch torque development (RTD), and range of motion of the plantar flexors. The authors found a decrease on the peak torque in all conditions which however did not differ from the control trial and concluded that static stretching may not be detrimental on performance. It is also interesting that the decrement in the peak torque returned to baseline 10 min post stretching in the 2 min stretch condition.

Nevertheless, numerous studies (Fowles et al. 2000; Cramer et al. 2005; Weir et al. 2005) pointed out that stretch-induced strength loss can be expected when passive stretches of long (10–30 min) duration are applied to the MTU. Other researchers (Knudson and Noffal 2005; Winchester et al. 2009; Brandenburg 2006) used shorter durations (10–60 s) of passive stretches in order to simulate the stretches used in the daily practice. For example Knudson and Noffal (2005) found that 20–40 s of passive stretches are sufficient to reduce the isometric grip strength in a logarithmic scale shape. Winchester et al. (2009) applied 1–6 bouts of 30 s passive stretches to the hamstrings and found out that the one repetition maximum (RM) was impaired (−5.4 %) after the first 30 s of passive stretch and continue dropping (12.4 %) until the last session. In another study, Brandenburg (2006) used two stretching protocols of 15 and 30 s duration and tested the isometric, concentric, and eccentric muscle actions. The author found that in all tested activities there was a significant (*p* < 0.05) decrement in force between the pre and post trials but there was no interaction effect across the different stretch durations. Although in those aforementioned studies the passive stretch duration used is similar to our study, the results differs. This could be explained by the different muscle groups the authors studied or the different experimental protocols utilized. A varying effect on different muscle groups was reported in a previous study by Power et al. (2004) who found a significant decrease in the isometric torque of the quadriceps muscle but not in the plantar flexors muscles after static stretch. Those differences were attributed to the individual fiber (slow–fast twitch) distribution. The effect of methodology (e.g. additional isometric contractions) can be observed in different studies. Knudson and Noffal (2005) repeatedly applied isometric contractions and passive stretches to the wrist flexors. The authors found significant differences compared to the control group after 40 s of combined stretch and isometric contractions. In a comparable procedure, Kay and Blazevich (2009b) performed six isometric MVCs and subsequently found a decrement in the concentric plantar flexor moment, Achilles tendon stiffness, and neuromuscular activity. However, a following passive stretch regime of 3 × 60 s did not significantly alter any of the measures. This indicates that repeated isometric contractions alone can induce changes in the mechanical properties of the MTU similar to the study by Knudson and Noffal (2005). Similar concerns about the effect of repeated muscular contractions on the performance can be raised also on the study of Winchester et al. (2009) where the onset of the stretch-induced strength loss was subject of the study. The authors found that 30 s of static stretch was a sufficient stimulus to induce a force decrease in a one repetition maximum trial of the knee flexors. As a consequence the authors suggested that athletes attaining maximum force should avoid static stretch prior to exercise. However, the determination of the 1-RM was achieved by multiple trials before reaching the final load. Therefore, this experimental procedure may also be affected through the—prior to test—multiple muscle contractions and could lead to erroneous results.

### Passive stretch

In order to identify any mechanical differences of the tendon and aponeurosis induced by the static stretch, we calculated an estimate for the passive stiffness of the elastic structures from the maximum achievable knee flexion position to the position where zero torque was reached. There was a limitation on the maximum resistive torque that could be achieved because of the technical constraints in the motion of the lower limb. Nevertheless, this technical restriction provided also a fix position at maximum achievable knee flexion for the pre–post trial, and hence any alteration of the mechanical properties of the tendon and aponeurosis could have been detected as a shift of the joint angle at zero torque point. A similar method was used by Nordez et al. (2010) where the authors examined the effect of static vs. dynamic stretching on changes of the joint angle at various levels of torque. With this method we overcame the constraints implied by the limited range of the knee joint during the passive motion and since we did not calculate the stiffness at the maximum passive torque the alternative approach (measuring a shift in the joint angle–torque relation) could also give an indication of alterations in the mechanical properties of the VL tendon and aponeurosis.

The results of our study showed (Table 1) that the knee joint angle range did not alter between and within all stretching groups indicating that the stretch stimulus was not sufficient to induce changes in the mechanical characteristics of the VL tendon and aponeurosis. Our results correspond with the findings provided by Nakamura et al. (2013) where the authors applied 1 min static stretch to the gastrocnemius muscle–tendon unit and found no alteration of the passive muscle tendon junction displacement and also the passive torque. Further repeatedly stretching, with 1 min interval, showed that a significant decrease of passive torque and an increase of the MTJ displacement from the 2 to 5 min of the applied static stretch. Muir et al. (1999) also reported that a 4 × 30 s passive static stretch intervention did not negatively affect the resistive torque of the plantar flexors.

Nevertheless, contrary results are also present; Ryan et al. (2009) stated that already 2 × 30 s of static stretch can change the mechanical properties of the plantar flexors muscles when determining the minimum duration of stretch that could alter musculotendinous stiffness. The different results in the present and the aforementioned study could be due to the different technic used to achieve the changes in the mechanical characteristics since Ryan et al. (2009) used the constant torque method while we performed the constant joint angle method. It is documented (Herda et al. 2011, 2014) that the musculotendinous stiffness can be altered when constant torque is applied rather than constant joint angle during the stretch program. Therefore, the small stretch duration combined with the constant joint angle technique used in our study and also highly probable in sport practice indicates that it is not likely to induce changes in the musculotendinous stiffness.

### Jumping performance

The lack of impairment in the force generation mechanism and the absence of alteration in the mechanical properties of the VL tendon and aponeurosis were possibly reflected in the jump performance. In both jumping tasks there was no significant main effect indicating that the stretch volume applied in this study was not sufficient to alter the jumping performance. It was postulated in numerous studies that acute passive static stretch induce the performance through neural inhibition (Avela et al. 2004) or alterations on the mechanical and morphological properties of the muscle tendon unit (Ryan et al. 2008a) which is suspected to further reduce the rate of force development that is being transferred to the bones (Power et al. 2004).

However, there are mixed results in the literature according the effect of passive stretch on jumping performance. For example, our result is partially in accordance to the findings of Power et al. (2004) who reported no influence of static stretch on jumping performance. They applied an overall total static stretch duration of 4.5 min per muscle group which was 4.5 times higher compared to the maximum stretch time in this study. Power et al. (2004) found a significant decrease (9.5 %) of MVC force that was associated to the reduced ITT (5.4 %) and therefore concluded that neurological effects could have caused that diminishing outcomes. Interestingly, those decrements were not present in the jumping performance and therefore authors further hypothesized that mechanical factors like the tendon and aponeurosis elasticity could have influenced the task.

On the contrary, Behm and Kibele (2007) reported a decrement in jump height after different intensities (50–75 and 100 % of point of discomfort) of static stretch. The authors showed that in various jumping tasks (drop, squat, and countermovement jump) there was a significant performance decrement which ranged from 3.6 to 5.7 %. The authors identified the intensity of stretch as main factor for the diminishing effect on the jump performance while the low duration of the static stretch was kept constant throughout the experiment. Similar to our study they stretched three muscle groups (hamstrings, quadriceps, and plantar flexors) of both limbs prior to jumping test. Instead of examining the ROM as done by Behm and Kibele (2007), we focused on the mechano-morphological properties of the VL tendon and aponeurosis. Given that, we can assume that two main factors could contribute to these differences between experiments. One may be the different overall duration (max 60 vs. 120 s) of static stretch on a single muscle group and the second may be the stretching mode (4 × 30 s) applied in the study of Behm and Kibele (2007). In the literature it is demonstrated that the passive stretch duration of 30 s is effective to alter the range of motion of the lower leg (Bandy et al. 1997), but also the 60 s interval is accepted in the scientific community as a static stretching interval (Kay and Blazevich 2009a, b) in order to detect effects of static stretching on neuromuscular activity or muscle–tendon mechanical and morphological properties. Therefore, it was expected (Simic et al. 2013) that already a stretch stimulus over 45 s would be adequate to induce changes in the mechanical morphological characteristics of the lower extremities and furthermore affect the jumping performance. Another point to consider is the time which passes from stretching to the jumping task. Various researchers (Ryan et al. 2008a, b; Mizuno et al. 2013) pointed out a dose and time-dependency of static stretch with alterations of the functional parameters of the muscle–tendon unit. A possible impairment of the force generating mechanism could be restored within 10 min post stretch (Mizuno et al. 2013) and therefore the results might be erroneous. Hence, care must be taken in future experiments accounting for the rest period used between the stretch regime and the measuring task.

## Limitations

We did not account for the inevitable joint rotation during the MVC effort (Arampatzis et al. 2006) and therefore the calculated VL tendon and aponeurosis elongation is probably overestimated. It was pointed out (Arampatzis et al. 2006) that the elongation of the VL tendon and aponeurosis was ~0.5 mm deg^−1^ due the passive rotation of the knee joint and also depending on the measurement point on the VL. This systematic error was present in both pre and post measurements. Since the exerted torque and the measuring point of the elongation of all groups did not differ we can assume that the overestimation of the elongation would be similar in both instances and therefore it would not affect the main results of this study.

Also, important for the assessment of the effects of static stretch on the mechano-morphological properties of the musculotendinous structures is the relation between magnitude of the applied load and time to restore the possible detrimental effects. It has been previously addressed that decrements in tendon stiffness were restored to base-line levels after a short duration. For example, Mizuno et al. (2013) demonstrated that 5 min of static stretch induces decrements in the tendon stiffness that returned to previous state within 5–10 min. Others (Ryan et al. 2008a) found a dependence of tendon stiffness restoration on the duration of static stretch. The authors showed that the low volume (2 min) of static stretch induced a decrement in musculotendinous stiffness (MTS) that returned to base-line levels within 10 min and those alterations of the MTS following moderate passive stretch duration (4–8 min) returned after 10–20 min to the initial state. It is therefore reasonable to speculate that the low volume static stretch used in our study would possibly affect the mechano-morphological properties of the VL tendon and aponeurosis but those effects could possibly have returned to the baseline earlier than in the aforementioned studies and as a consequence could not be detected within the present time frame. The time to measure could be also affected by the randomization of the stretches in both muscles and legs. In contrast to a single measure experimental procedure, we assessed various parameters of the right VL-MTU although through the randomization procedure the target MTU could be stretched at any time (first to last) given an estimated time gap from 2 to 10 min until the measuring session. This time gap is getting greater when assessing the subsequent jumping performance (Fig. 4). It is therefore reasonable to assume that probable alterations of the mechano-morphological properties of the MTU could be restored in that remained time as previous studies have shown (Ryan et al. 2008a; Mizuno et al. 2013). However, such a return to base-line levels of the mechanical properties can also be assumed when static stretch is practiced in recreation and competitive activities due to similar time frames (5–10 min) from stretching to the actual task. Taking into account the low volume of static stretch practiced in those activities, we can speculate that effects would be marginal or even not detrimental since they possibly last only for a short period of time. Nevertheless, further studies need to be conducted in order to establish the dose–time relation of the low volume static stretch on the muscle tendon unit.

The results could also have been influenced by the mix gender population used in this study. The coefficient of variation in the examined parameter lay between 16 and 26 % possibly because of the different sports practice background and the gender differences. Therefore, we cannot exclude that results are different in subjects with specific age, gender, or training background. Nevertheless, a separate analysis including only the male subjects in this study resulted in the same outcome (data not presented).

## Conclusions

Concluding, the overall stretch duration used in the present study did not negatively influence jumping performance or isometric muscle force. Besides the short stretch duration, the time delay between stretching and physical performance, which was between two and ten minutes in this study, might have also influenced the results. Taking into account the time delay due to the different movements, and tasks incorporated in the warming up routine by athletes prior to high strenuous exercise we cannot confirm the deleterious effect of static stretching on performance suggested in previous studies (Behm and Kibele 2007; Young and Behm 2003; Simic et al. 2013).

## Abbreviations

ANOVA: Analysis of variance
CMJ: Countermovement jump
MTS: Musculotendinous stiffness
MTU: Muscle tendon unit
MVC: Maximum voluntary contraction
POD: Point of discomfort
RPM: Revolutions per minute
SJ: Squat jump
VL: Vastus lateralis
